# Analysing detection gaps in acoustic telemetry data to infer differential movement patterns in fish

**DOI:** 10.1002/ece3.7226

**Published:** 2021-02-10

**Authors:** Michael J. Williamson, Emma J. Tebbs, Terence P. Dawson, David J. Curnick, Francesco Ferretti, Aaron B. Carlisle, Taylor K. Chapple, Robert J. Schallert, David M. Tickler, Xavier A. Harrison, Barbara A. Block, David M.P. Jacoby

**Affiliations:** ^1^ Department of Geography King’s College London London UK; ^2^ Institute of Zoology Zoological Society of London London UK; ^3^ Department of Fish and Wildlife Conservation Virginia Tech Blacksburg Va USA; ^4^ Hopkins Marine Station Stanford University Pacific Grove CA USA; ^5^ School of Marine Science and Policy University of Delaware Lewes DE USA; ^6^ Marine Futures Lab School of Biological Sciences University of Western Australia Perth WA Australia; ^7^ Centre for Ecology and Conservation University of Exeter Penryn UK

**Keywords:** animal movement, biotelemetry, elasmobranchs, marine protected areas, network analysis, sharks, spatial and temporal segregation, sympatry

## Abstract

1. A wide array of technologies are available for gaining insight into the movement of wild aquatic animals. Although acoustic telemetry can lack the fine‐scale spatial resolution of some satellite tracking technologies, the substantially longer battery life can yield important long‐term data on individual behavior and movement for low per‐unit cost. Typically, however, receiver arrays are designed to maximize spatial coverage at the cost of positional accuracy leading to potentially longer detection gaps as individuals move out of range between monitored locations. This is particularly true when these technologies are deployed to monitor species in hard‐to‐access locations.
2. Here, we develop a novel approach to analyzing acoustic telemetry data, using the timing and duration of gaps between animal detections to infer different behaviors. Using the durations between detections at the same and different receiver locations (i.e., detection gaps), we classify behaviors into “restricted” or potential wider “out‐of‐range” movements synonymous with longer distance dispersal. We apply this method to investigate spatial and temporal segregation of inferred movement patterns in two sympatric species of reef shark within a large, remote, marine protected area (MPA). Response variables were generated using network analysis, and drivers of these movements were identified using generalized linear mixed models and multimodel inference.
3. Species, diel period, and season were significant predictors of “out‐of‐range” movements. Silvertip sharks were overall more likely to undertake “out‐of‐range” movements, compared with gray reef sharks, indicating spatial segregation, and corroborating previous stable isotope work between these two species. High individual variability in “out‐of‐range” movements in both species was also identified.
4. We present a novel gap analysis of telemetry data to help infer differential movement and space use patterns where acoustic coverage is imperfect and other tracking methods are impractical at scale. In remote locations, inference may be the best available tool and this approach shows that acoustic telemetry gap analysis can be used for comparative studies in fish ecology, or combined with other research techniques to better understand functional mechanisms driving behavior.

## Graphical abstract

Financial and logistical constraints may lead to imperfect in‐field acoustic receiver array designs which can result in acoustic telemetry data with long detection gaps. We present a novel gap analysis of telemetry data to help infer differential movement and space use patterns where acoustic coverage is imperfect and other tracking methods impractical

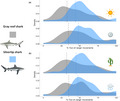

## 1 INTRODUCTION

Biologging and biotelemetry are now ubiquitous in aquatic ecology, revealing important insight into the movement patterns of a broad spectrum of species (Block et al., 2011; Carrier et al., 2018; Hussey et al., 2015). For example, geolocations from pop‐up satellite archival tags (PSATs) can be used to reconstruct estimated tracks of tagged animals that rarely come to the surface. Although satellite telemetry has greatly advanced our knowledge of aquatic species (Hammerschlag et al., 2011; Hussey et al., 2015), it can be constrained by high costs, battery life, and the low spatial accuracy of estimated positions (Ferreira et al., 2018). Acoustic telemetry is a popular alternative for monitoring the spatial ecology of aquatic species, particularly of those that have a tendency to be site‐attached (e.g., reef fishes), as it can prove cheaper and enable the monitoring of wildlife over longer time periods (Donaldson et al., 2014; Heupel et al., 2018; Hussey et al., 2015). However, there are a number of trade‐offs to consider when establishing acoustic arrays that are influenced by scale, field logistics, and habitat type; consequently, the spatial configuration of receiver arrays is often designed to maximize spatial coverage at the cost of positional accuracy leading to numerous blind spots (Heupel, Kessel, et al., 2018; Kessel et al., 2014). This can potentially lead to longer detection gaps as individuals move out of range between monitored locations (Kessel et al., 2014), and limit investigations into certain ecological questions, such as relative space use between species.

Acoustic telemetry is increasingly becoming an important tool for researchers and is used across a wide range of aquatic species and environments (Abecasis et al., 2018; Donaldson et al., 2014). Previously, it has been usefully employed to inform spatial management (Heupel, Kessel, et al., 2018), in particular, in assisting the designation and evaluation of marine protected areas (MPAs) (Carlisle et al., 2019; Espinoza et al., 2015; Knip et al., 2012). Acoustic telemetry, however, is primarily used not only to measure or infer multiple aspects of ecology in aquatic wildlife (Hussey et al., 2015; Mourier et al., 2018), such as social structuring (Guttridge et al., 2011; Jacoby et al., 2016; Wilson et al., 2015) and individual social preferences (Findlay et al., 2016), but also to investigate spatiotemporal distribution and movement dynamics (Heupel, Kessel, et al., 2018; Heupel et al., 2019; Jacoby et al., 2012; Williams et al., 2018).

Investigating changes in movement patterns over time is important for understanding how species can influence one another, such as how predators impact prey through predation (Speed et al., 2010). Knowledge of these mechanisms is important as this can lead to top‐down effects through mortality and antipredator behavior, resulting in changes to prey communities and species abundance (Creel & Christianson, 2008; Ferretti et al., 2010; Heithaus et al., 2008; McCauley et al., 2012). Temporal changes in movement patterns (e.g., seasonal or diurnal) of aquatic wildlife may also have bottom‐up effects by impacting nutrient cycle timings in marine ecosystems, such as coral reefs (Williams et al., 2018). Consequently, the distribution and timing of acoustic detection data can be extremely informative at daily, monthly, seasonal, and annual scales. However, there is also important information contained within the absences between detections that are often overlooked. Thus, it is important to identify not only the frequency and periodicity of movements to areas of interest (e.g., feeding and breeding areas, and resting refugia), but also the time taken for movements between these and other areas (Calabrese & Fagan, 2004), specifically, the periods when they are not being detected.

Network analyses of movements derived from acoustic telemetry are becoming more commonplace for exploring not just the spatial but also the temporal patterns of movement within acoustic detection data (Jacoby & Freeman, 2016; Jacoby et al., 2016; Mourier et al., 2018). Typically, network analyses of acoustic telemetry data often ignore the gaps between detections. These gaps can be informative for inferring the length of time taken between movements, and as a proxy of tortuosity in fish species that must constantly swim for example, as the longer the duration between two points the greater the tortuosity of the movement is likely to be. Therefore, the analysis of detection gaps from acoustic telemetry data can be useful for investigating coarse‐scale behavior and its associated timings. For example, gaps might be used to inform the likelihood of fish moving out of marine protected areas (MPAs) into unprotected waters, where they may be vulnerable to exploitation from commercial fisheries (Carlisle et al., 2019). These gaps can also be used to estimate the timings of ontogenetic habitat shifts, when individuals begin leaving nursery areas for longer periods (Poulakis et al., 2013), as well as for more accurately determining the timings and thresholds to define residency events for spatial distribution and movement analyses (Chapman et al., 2019).

The British Indian Ocean Territory (BIOT) is a large, remote archipelago declared a “no‐take” MPA in 2010, the reefs of which are home to multiple elasmobranch species (Koldewey et al., 2010; Sheppard et al., 2017). In this study, we utilize an extensive acoustic tracking data set from this region to present an approach to monitor coarse‐scale movements of sympatric reef shark species in the absence of full receiver coverage or sufficient satellite telemetry data to determine broader (pelagic) activity. Gray reef sharks (*Carcharhinus amblyrhynchos*) and silvertip sharks (*Carcharhinus albimarginatus*) were used as model species for this study as they are the most abundant large predator species in the BIOT MPA, and often co‐occur with the potential for competition for resources (Carlisle et al., 2019; Curnick et al., 2019). Our aim was to (1) develop and test an approach for identifying informative detection gaps between movements from acoustic telemetry data; and (2) combine this approach with information‐theoretic modeling to analyze and assess the potential of detection gaps to investigate differential movement patterns and segregation in sympatric species.

## 2 MATERIALS AND METHODS

### 2.1 Data collection and study site

**Figure 1:**
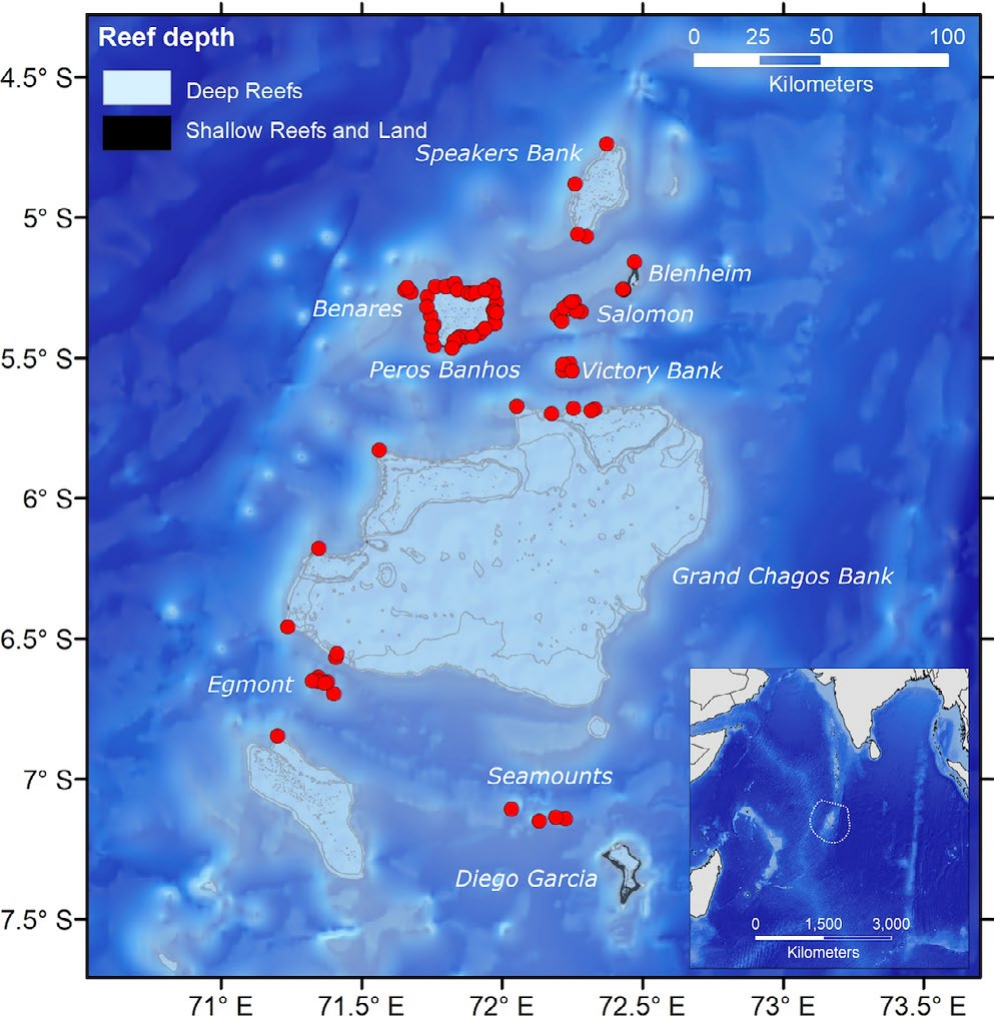
Acoustic array in the BIOT MPA with the locations of 93 acoustic receivers shown in red, adapted from Carlisle et al. (2019). Insert shows the location of the BIOT MPA in the Indian Ocean, with the exclusive economic zone (EEZ) and MPA boundary indicated by the dotted line. Gray lines show the contours of major submerged geographic features. Shallow reefs are <20 m in depth, with deep reefs between 20 and 100 m in depth

Acoustic telemetry data were collected in the BIOT MPA between 2014 and 2018. Throughout the archipelago, there have been situated up to 93 permanent and temporary acoustic receivers (VR2W, VR4‐UWM, VR4G, and VR2AR receivers; Vemco Inc., Nova Scotia, Canada), as configured in Figure 1. The BIOT MPA is characterized by numerous small islanded atolls with submerged banks and reefs, with depths of 1,000 m or more separating each atoll or reef system (Sheppard et al., 2013). Acoustic receivers in the BIOT MPA are mainly based on areas accessible to divers, such as coral reef systems, with few receivers covering the deep pelagic waters of the region. In addition, the considerable size of the MPA [640,000 km^2^ (Sheppard et al., 2013)] limits the ability and spatial resolution of monitoring wildlife movements in this region. These factors result in the array having some significant blind spots between and within some of the larger reef systems. Close‐up maps of the receiver deployments at three of the most well‐monitored reef systems within the BIOT MPA, and an assumed 500 m detection range, can be found in Appendix 1: Figures S1–S3. The BIOT MPA receiver array was initially started in 2013, and expanded throughout subsequent years [for more information, see Carlisle et al. (2019) and Jacoby et al. (2020)], covering a perimeter of 700 km and an area of 25,500 km^2^ within the MPA, for the detection of acoustically tagged marine fauna. Of the 93 receivers, 82 are in depths of 45 m or less. All receivers were situated far enough apart to avoid overlap in their detection range, with mean distance to closest receiver being 2.15 km, with a range of 0.55–4.57 km (the frequency distribution of interreceiver distances can be found in Appendix 1: Figure S4). Although range testing has not been undertaken for this array due to financial and logistical constraints of vessel time in the BIOT MPA, other studies conducted around coral atolls in the Indian Ocean using the same or similar equipment have found detection ranges between 300 and 500 m (Field et al., 2011; Forget et al., 2015; Govinden et al., 2013; Speed et al., 2011).

Gray reef sharks (*Carcharhinus amblyrhynchos*) and silvertip sharks (*Carcharhinus albimarginatus*) were used as model species for this study as they are the most abundant large predator species in the BIOT MPA, appear sympatrically across the region and they are both a target for illegal fishing activity that continues to plague the MPA (Tickler et al., 2019). Importantly for our methodology, both species are ram ventilators and have to keep moving in order to breathe (Skomal et al., 2007). Data were collected on shark detections between 2014 and 2018. In total, our data comprise 102 gray reef and 75 silvertip sharks tagged with acoustic transmitters across nine different locations following the methodology described by Carlisle et al. (2019)**.** Of tagged gray reef sharks, 76 were female and 26 were male, and for the tagged silvertip sharks, 44 were female and 31 were male. As in previous studies (Barnett et al., 2012; Espinoza, Lédée, et al., 2015), silvertip sharks (mean total length, TL = 123.56 cm ± S.D. 19.14) were on average slightly larger than gray reef sharks (mean TL = 119.15 cm ± S.D 18.07). Detailed metadata for each tagged individual can be found in Appendix 1: Table S1. Tags were configured to transmit an acoustic “ping” containing a unique ID code with a nominal delay of 60–180 s for the duration of their battery life (~10 years), providing a long‐term time series of detection data. Receivers were downloaded and serviced annually at the same time each year (March–May).

### 2.2 Movement classification

**Figure 2:**
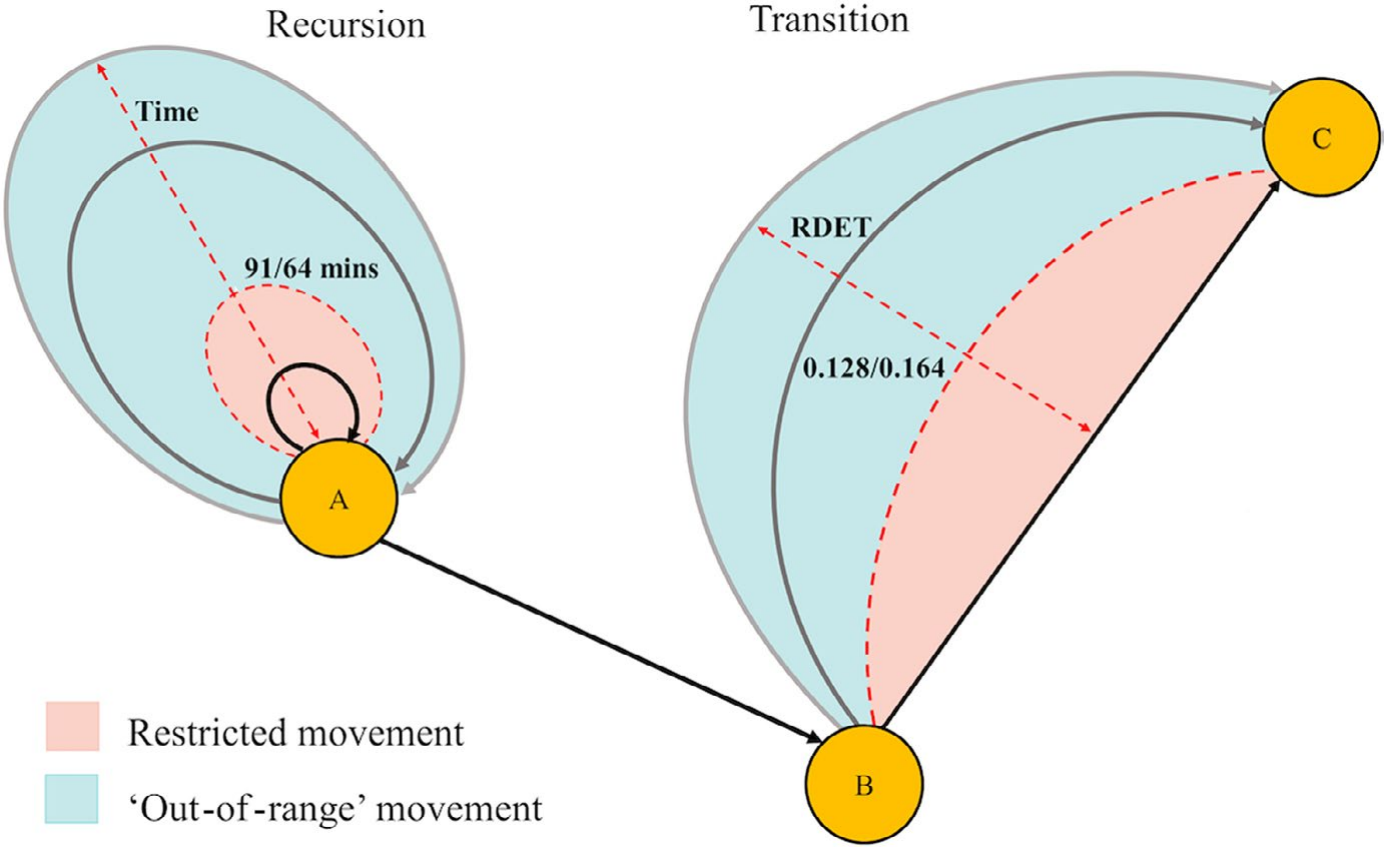
Schematic describing designation of “restricted” and “out‐of‐range” movements. Black and gray arrows indicate a movement either to and from the same point (recursion), or between two points (transition). Time between detections for recursions, and relative deviation from expected time (RDET) for transitions, is represented by length, curvature, and color of the arrow. As time and RDET increase, length and curvature increase, and color gets lighter indicating less‐directed movement. Red dashed line indicates our cutoff detection gap (91 min for gray reef sharks and 64 min) for silvertip sharks for recursions and RDET (0.128 for gray reef sharks and 0.164 for silvertip sharks) for transitions

Acoustic telemetry generates presence‐only, time‐series information for individuals carrying uniquely coded transmitters across receivers often deployed as an array and is commonly used to monitor the attendance and residency of individuals/species at specific sites (Heupel et al., 2006; Vianna et al., 2014). Network analysis was used here to define and distinguish between two different types of shark movement within coral reef systems from acoustic telemetry data. A detection gap is the length of time between two consecutive detections from the same individual. Using a movement network approach, this can be when an individual leaves one receiver and arrives at another in a new location, known as a “transition” (Jacoby et al., 2012). Alternatively, an animal might leave a receiver, move out of detection range, and then return to the same location, a “self‐loop” in network parlance, but here called a “recursion.” These two movement types were used to assess “restricted” and “out‐of‐range” activity, where “restricted” activity is defined as on‐reef movements within the acoustic array, and “out‐of‐range” activity, defined as wider, off‐reef movement activity. In order to investigate differential movement patterns, classification of “restricted” and “out‐of‐range” activity was, therefore, inferred based on the duration of transitions and recursions (Figure 2), before being tested as a binary response variable in subsequent models using our empirical example.

All analyses were conducted in R version 3.6.0 (R Core Team, 2019). To avoid false detections from unknown animals in our study system, only detections from animals with known ID codes were used for the analyses. For recursions, detection gaps of less than six minutes (minimum of two detections) were removed from the data. This was undertaken as an initial filter to ensure a recursion had taken place, rather than an animal had stayed in the same location but a detection had been missed. In addition, this reduces the possibility of false positives from the recursion data set, as any sequential detections at the same receiver quicker than the repeat rate are removed (Simpfendorfer et al., 2015). To avoid subjective methodologies, such as visual assessment with histograms, classification of “restricted” and “out‐of‐range” movements was conducted using an optimal classification method, where similar data values were placed in the same class by minimizing an objective measure of classification error, such as numerical mean (Slocum et al., 2009). Time differences for recursive movements per species were log‐transformed to normalize the data, and the “classIntervals” function in the *classInt* package (Bivand et al., 2019) was used to calculate thresholds between “restricted” and “out‐of‐range” movements. The Fisher algorithm was used, which determines thresholds by minimizing the sum of absolute intraclass mean variance, as well as maximizing interclass mean variance (Fisher, 1958; Slocum et al., 2009). This resulted in a threshold of 91 min for gray reef sharks and 64 min for silvertip sharks for “restricted” activity, beyond which it was assumed that the shark had conducted an “out‐of‐range” movement.

Transitions were subject to a separate filtering process. Unlike recursions, no initial filter was required for transitions as the detection of an individual on one receiver followed by another receiver is immediately indicative of a movement from one location to another. Temporal gaps in the detection data for any given pair of receivers were informed by both the distance and species‐specific minimum sustainable swim speeds (0.69 m/s for gray reef sharks and 0.73 m/s for silvertip sharks) (Jacoby et al., 2015). For example, the predicted transition duration of a direct movement of a shark between two receivers, without deviation, would be the ratio between distance and speed. As such, by first calculating expected time for a transition using swim speeds and distance, the relative deviation from this expected time (RDET) between any pair of receivers was determined by dividing the expected transition time by the observed transition time. RDET values of >1 were movements faster than expected, and values of <1 were slower/more tortuous than expected (Figure 2). As high RDET indicates swim speeds much greater than expected, RDET values greater than 5 (5 times expected speed) were removed to remove the chances of false detections.

For transitions, log‐transformed RDET values were calculated for both species, and as with recursions, the same optimal classification method for determining thresholds was used (Slocum et al., 2009). Movement values greater than the threshold value of 0.164 for gray reef sharks and 0.128 for silvertip sharks were determined as “restricted,” with values less than the thresholds determined as “out of range.” Animals rarely travel in straight lines and often vary in their tortuosity depending on factors such as resource use, habitat quality, competition, and predation (Fahrig, 2007; Gurarie et al., 2009; Roshier et al., 2008). These thresholds of 0.164 and 0.128 are, therefore, very conservative, to allow for a tortuous movement to occur and still be classified as “restricted” in each species. Finally, recursions and transitions were combined so that every movement was categorized as a binary response (“restricted” = 0, “out of range” = 1) (Figure 2). Our conservative classification thresholds allow for missed detections and movements to still be classified as “restricted.” As both species are ram ventilators (Skomal et al., 2007) and are therefore unable to rest motionless, we assume that if an animal is absent from the array for longer than the determined threshold, that it has left the reef region, rather than remaining in an area where there were no receivers or where there is poor detection efficiency. In addition, it is worth noting that transitional and recursive movements are dependent on the scale of the array. What would be termed two transitional movements in a fine‐scale array (from A to B to A) may be a recursion in an array with larger spacing between receivers. However, this does not impact our methodology as the two movements are combined.

### 2.3 Data analysis

To explore the influence of explanatory variables on “out‐of‐range” movements, an information‐theoretic approach was taken, which accounts for model selection uncertainty (Burnham & Anderson, 2002; Harrison et al., 2018). In recent years, information‐theoretic approaches have become a staple for modeling ecological systems, particularly those where explanatory models describing the system may have similar complexity and fit the data equally well, such as understanding the spatial distribution (Diniz‐Filho et al., 2008; Greaves et al., 2006; Rhodes et al., 2009), behavior (Garamszegi, 2011; Kavanagh et al., 2017), and anthropogenic impact on survival of wildlife populations (Aronson et al., 2014; Currey et al., 2009). To limit exploratory analyses, and prevent model overfitting, an *a priori* selection of variables and interactions based on previous research and theory was conducted (Dochtermann & Jenkins, 2011; Grueber et al., 2011; Harrison et al., 2018). Explanatory variables included in the model were “species,” “sex,” “size,” “season” (wet/dry), and “diel period” (day/night) (Andrews et al., 2009; DiGirolamo et al., 2012; Dudgeon et al., 2013; Espinoza, Lédée, et al., 2015; Heupel et al., 2019). As size had a non‐normal distribution, it was log‐transformed. The BIOT MPA is located near the equator and has a roughly 12‐hr day/night cycle. As such, day was designated from 0700 to 1900 and night from 1900 to 0700 following sunrise and sunset times obtained from https://www.timeanddate.com. The MPA experiences distinct Indian Ocean wet and dry seasons with wet season running from October to March and dry season from April to September (Sheppard et al., 2012). Seasonal variability is often greater than monthly variability in tropical ocean systems (Huang & Kinter III, 2002; Servain et al., 1985), and therefore, we deemed season a more biologically relevant driver of shark movement.

All variables used in the model were assessed for multicollinearity. Multicollinearity, which occurs when predictors in a multiple regression are highly correlated (McGowan et al., 2012), was assessed by producing a variance inflation factor (VIF) using the “check_collinearity” function in the *performance* package in R (Lüdecke et al., 2019). VIF measures the degree of multicollinearity in a regression model by providing an index of how much the variance of the model variables increases due to collinearity (O’brien, 2007). No evidence of collinearity was found, with all variables having a VIF ≤ 1.05, less than the critical threshold of 5.0 (see Appendix 1: Table S2) (McGowan et al., 2012; Welzel & Deutsch, 2011). As such, all a priori selected explanatory variables were included in the global model.

A global model was subsequently created using a generalized linear mixed model (GLMM) (family = binomial, link = logit) in the *glmmTMB* package (Brooks et al., 2017). To explore putative spatial and temporal segregation between gray reef and silvertip sharks, “restricted” versus “out‐of‐range” movements were included as a binary response variable and “species” was included as interaction term with all explanatory variables and individual ID as a random factor. As the likelihood of a movement between locations decays as a function of distance (Jacoby et al., 2020), receiver location was also included as an independent random factor. Residuals of the global model were checked for heteroscedasticity, and autocorrelation and data were checked for binomial distribution using the functions “resid,” “fitted,” and “acf” from the *stats* package (R Core Team, 2019) and found free from autocorrelation and heteroscedasticity of residuals (Appendix 1: Figure S5).

To generate the model set from the global model, the “dredge” function from the *MuMIn* package was used (Bartoń, 2009). Models in the set were ranked by small sample size Akaike information criterion (AICc) values (Burnham & Anderson, 2002; Grueber et al., 2011; Harrison et al., 2018). As inference using AICc can be made more reliable by removing models, which are more complex versions of others (Grueber et al., 2011; Richards, 2008), the “nested” function from the *MuMIn* package was used on the model selection table. If a single parsimonious model remains following these analyses, this model is fitted to the data. If no single parsimonious model subsequently results from the set and the weight of the best model is less than 0.9, model averaging is recommended (Grueber et al., 2011).

Parameter estimates indicate the change in probability of observing an “out‐of‐range” movement as the value for continuous predictor variables increases. Categorical predictor variables were compared to the categorical variable level used as the model baseline. Positive estimates indicate an increased probability of “out of range” and a decreased probability of “restricted” movements; negative estimates, the reverse. To assess the effect of the fixed effects on the model, and the combination of fixed and random effects (Johnson, 2014; Nakagawa & Schielzeth, 2013), marginal *R^2^* (R^2^m) and conditional *R^2^* (R^2^c) values were calculated, using “r.squaredGLMM” in the *MuMIn* package (Bartoń, 2009; Nakagawa & Schielzeth, 2013), and conditional models of the random effects, and their standard deviations, were extracted from the top model using the “ranef” function from the *lme4* package (Bates et al., 2015).

### 2.4 Model cross‐validation

To assess the predictive capabilities of our final model, analysis was conducted on 80% of the data. Cross‐validation of the model estimate values was conducted on the remaining 20% of data as confirmation of how well the selected model performed (Harrison et al., 2018). The “predict” function in the *glmmTMB* package was used to validate the expected outputs of the multimodel inference on the observed values from the reserved 20% of the data. Area under the receiver operating characteristic curve (AUC) values designates the probability that positive and negative instances are correctly classified (Siders et al., 2013). As such, AUC was calculated using the *pROC* package (Robin et al., 2011), as a threshold‐independent method to check the robustness of the model. An AUC value of greater than 0.5 indicates better than random performance (Jiménez‐Valverde, 2012, 2014; Swets, 1988).

## 3 RESULTS

### 3.1 Sample size

**Figure 3:**
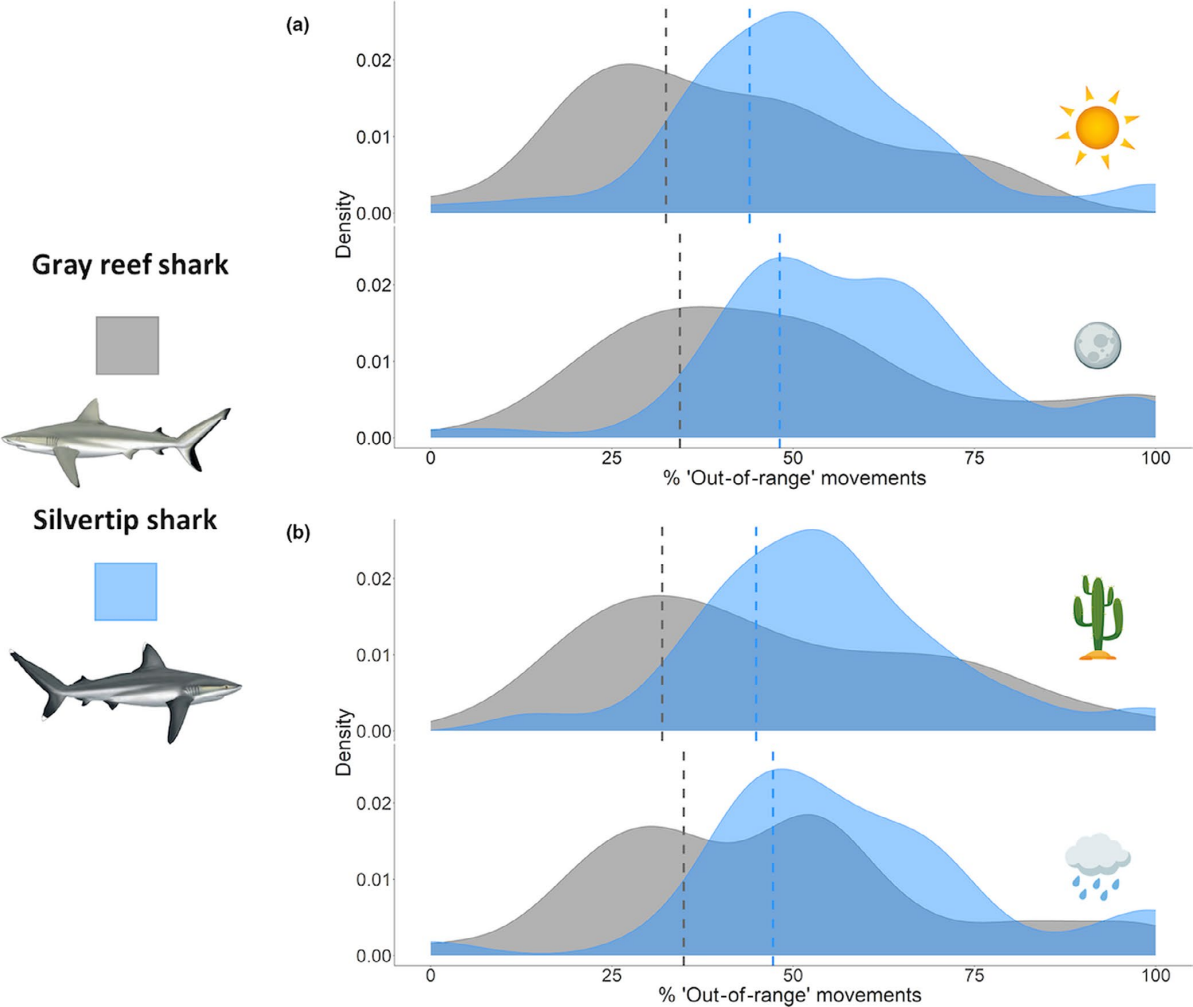
(a) Frequency density plots of gray reef shark (gray) and silvertip shark (blue) diel variance (a) and seasonal variance (b) in percentage “out‐of‐range” movements, with the sun, moon, cactus, and rain cloud indicating daytime, nighttime, dry season, and wet season “out‐of‐range” movements, respectively

Between January 2014 and December 2018, there were 206,619 movements (gray reef shark = 134,201, silvertip shark = 72,418), transitional and recursive, identified from 102 gray reef sharks and 75 silvertip sharks. From these movements, 129,292 were identified as “restricted,” and 77,327, “out of range.” Gray reef sharks conducted 67.0% and 33.0% % of “restricted” and “out‐of‐range” movements, respectively. Silvertip sharks conducted 54.3% and 45.7% of “restricted” and “out‐of‐range” movements, respectively. Mean “out‐of‐range” movements increased from 32.5% and 43.9% during the day to 34.4% and to 48.1% at night for gray reef sharks and silvertip sharks, respectively (Figure 3a). Mean “out‐of‐range” movements increased from 31.9% and 44.9% in the dry season to 34.9% and to 47.2% in the wet season, in gray reef sharks and silvertip sharks, respectively (Figure 3b).

### 3.2 Model analysis

Residuals of the global model were free from heteroscedasticity and temporal autocorrelation (Appendix 1: Figure S5). Following the dredge and nesting of the global model, one single parsimonious model was found. Although the weight of the top model was 0.25, as only a single model remained following “dredging” and “nesting,” model averaging was not undertaken. This model was fitted to the data, and the results from model analysis are presented in Table 1.

**Table 1:** Model averaging results following model selection for out‐of‐range movements

	Estimate	Std. error	CI		z value	*p* value
Intercept	−0.475	0.111	−0.693	−0.257	−4.27	.000
Diel period						
**Night**	**0.179**	**0.011**	**0.158**	**0.200**	**16.673**	**.000**
Season						
**Wet season**	**0.159**	**0.011**	**0.137**	**0.181**	**13.961**	**.000**
Species						
**Silvertip shark**	**0.449**	**0.111**	**0.231**	**0.667**	**4.037**	**.000**

Note

Estimates with standard error, 97.5% confidence intervals (CI), and associated *p* values are presented. Significant results are highlighted in bold.

**Figure 4:**
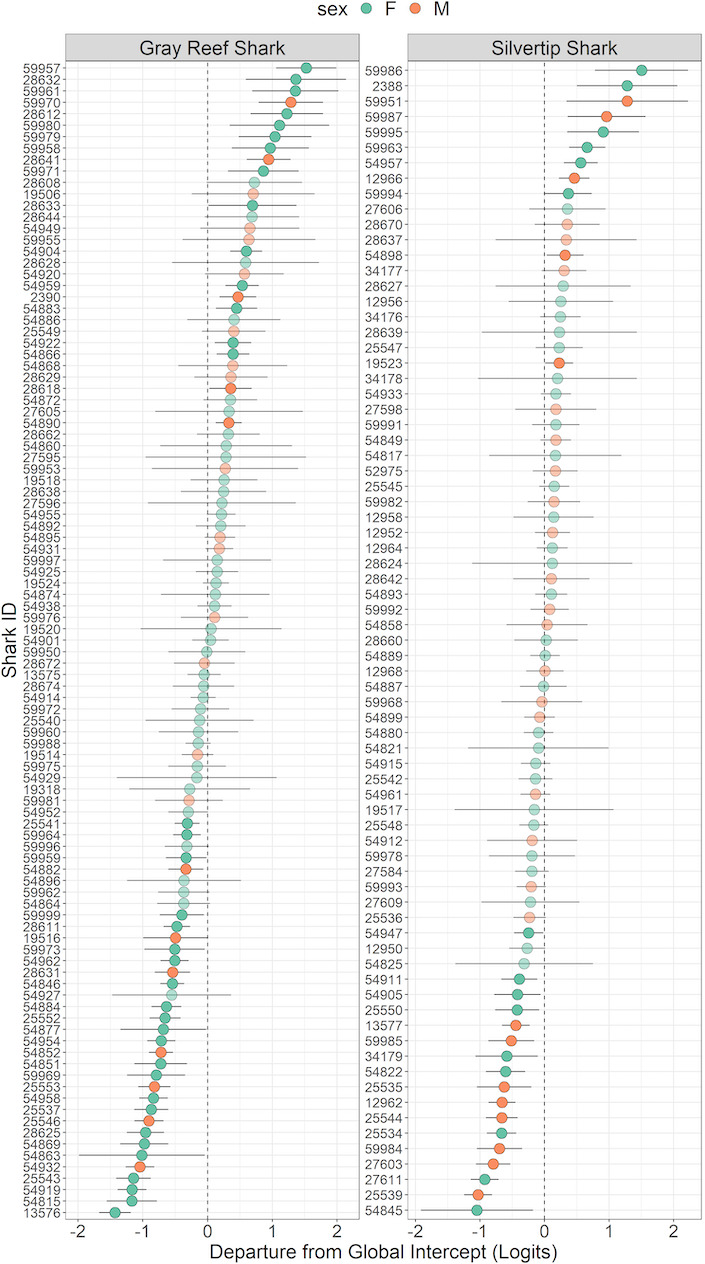
Plots of condition modes of random effects for individual gray reef and silvertip sharks. Departures from global intercept are plotted with 95% CIs (black bars). Individuals where CIs do not cross zero indicate average “out‐of‐range” movements significantly different than the average. Individuals conducting less than the average “out‐of‐range” movements have negative global intercept values, and those that conduct more have positive intercept values. Individuals are identified by species and sex

No interactions were included in the final model. Species, diel period, and season were significant predictors of “out‐of‐range” movements (Table 1). Silvertip sharks were overall more likely to undertake “out‐of‐range” movements compared with gray reef sharks (*p* < .001, Table 1), suggesting spatial segregation between the species. However, as both species still undertook regular “out‐of‐range” and “restricted” movements, this segregation was not discrete. “Out‐of‐range” movements were more likely to occur at night than during the day (*p* < .001, Table 1) (Figure 3a), and during the wet season than the dry season (*p* < .001, Table 1) (Figure 3b). The variance and standard deviation of the random factors on the logit scale were 0.43 and 0.65 for individual ID, and 0.43 and 0.66 for receiver location, respectively. Marginal *R^2^* (R2m) was 0.02 and conditional *R^2^* (R2c) 0.22, suggesting high individual variation in both species (Figure 4). Results from conditional models of the random effects and their standard deviations showed that 48% of gray reef sharks had significantly different “out‐of‐range” movements from the intercept (Figure 4). For silvertip sharks, 37% of individuals had significantly different “out‐of‐range” movements relative to the intercept. Model validation results calculated an AUC value of 0.68.

## 4 DISCUSSION

Here, we developed a new approach, which utilizes gaps in detections from acoustic telemetry to infer presence or absence from regions of interest, such as in our case, coral reef systems. Currently, analyses using GLMMs on acoustic telemetry time‐series data typically underuse information from timings and periodicity of detection gaps. However, as seen here, the analysis of detection gaps has the potential, albeit at coarse scales, to identify both spatial and temporal differences in movement in sympatric marine species, as well as high individual variation in movements in both species. As such, this method could potentially enhance our understanding of the organization and spatial distribution of aquatic wildlife, in a variety of environments, from telemetry data, where coverage is far from complete and in the absence of more accurate movement data for large numbers of animals, which can be prohibitively expensive using satellite telemetry.

Gray reef and silvertip sharks in the BIOT MPA had significant differences in “out‐of‐range” movements, which can be inferred as wider, off‐reef movement activity. Overall, silvertip sharks were more likely to undertake these potential wider, “out‐of‐range” movements than gray reef sharks. These results suggest spatial segregation between these species, with gray reef sharks, probabilistically, more likely to inhabit reef‐based areas, while silvertip sharks were more mobile and conducted more widespread movements (Figure 3). These results extend previous research describing variable patterns of movement and activity in both gray reef sharks and silvertip sharks globally (Espinoza et al., 2015; Papastamatiou et al., 2018; Vianna et al., 2013). In addition, this also supports previous research in the BIOT MPA, which found that silvertip sharks had higher mobility, larger activity spaces, and lower reef residency compared with gray reef sharks, which had small activity spaces (Carlisle et al., 2019; Jacoby et al., 2020).

Our results indicate that although there is spatial segregation between the species, there is probable overlap between the two in the areas they reside (Figure 3). This supports evidence from stable isotope data from this region, with each species utilizing both reef and pelagic areas for foraging, but with gray reef sharks obtaining 78% of their biomass from reef resources, but silvertip sharks only 60% (Curnick et al., 2019), as well as movement data from the BIOT MPA showing that, despite the wider dispersal in silvertip sharks, there are overlapping activity spaces between the two species (Carlisle et al., 2019; Jacoby et al., 2020).

Prior research on patterns of movements between coral reef‐associated elasmobranch species has been limited but primarily focus on differences in space use (Heupel et al., 2018, 2019; Papastamatiou et al., 2006). To date, temporal aspects of segregation are rarely considered (Bracis et al., 2018; McClintock et al., 2014). However, temporal patterns of movement, such as diel stage and season, are common in multiple elasmobranch species (Dudgeon et al., 2013; Heupel et al., 2004; Papastamatiou & Lowe, 2012). Our method found seasonal variance in probable wider “out‐of‐range” movements and diel variance between species. This suggests temporal segregation of movements between the two species, with silvertip sharks more likely to conduct “out‐of‐range” movements at night, and gray reef sharks showing smaller diel change (Figure 3a).

In addition, the model variance results, low marginal *R^2^* values from our fixed effects relative to our conditional *R^2^* values, and the results from conditional models of the random effects suggest that, in both gray reef sharks and silvertip sharks, interindividual variability plays an important role in explaining the probability of “out‐of‐range” movements (Figure 4). This suggests that within a species, some individuals have a tendency to be more wide‐ranging than others, which have limited dispersal ranges (often termed “partial migration”).

Although the low *R^2^* does mean we should interpret our particular results with some caution, our AUC values indicate that the model is a decent representation of our system (Jiménez‐Valverde, 2014; Swets, 1988). In addition, the use of *R^2^* to evaluate linear regressions of binary responses can be misleading (Cox & Wermuth, 1992; Mittlböck & Heinzl, 2001). Low *R^2^* values in ecological systems are not uncommon and can be expected when using linear regressions of binary responses as, in empirical research, it may be improbable to find explanatory variables that give predicted probabilities close to 0 or 1 (Ash & Shwartz, 1999; Cox & Wermuth, 1992; Mittlböck & Heinzl, 2001). Although they may prevent the use of the model in model predictions, they can, however, still be of use for describing processes within model systems (Alexander et al., 2015; Ash & Shwartz, 1999; Colton & Bower, 2002; Novak & MacEvoy, 1990). As such, this approach has shown that not only can gaps in detections be used to help interpret and support other types of data, but, despite the low *R^2^* values from the model, it also has the potential to provide insight into ecological mechanisms in environmental systems at coarse scales, such as the differences in spatiotemporal movements that underpin reef predator sympatry.

Because acoustic telemetry only measures presence, array design and detection ranges can significantly impact results obtained using this technology (Carlisle et al., 2019; Kessel et al., 2014). An inability to detect an animal could be due to the animal leaving the study area, or because it moved out of detection range (Heupel, Kessel, et al., 2018), and consequently might result in some misdesignated movements in this study. Detection ranges for the region vary between 300 and 500 m (Field et al., 2011; Forget et al., 2015; Govinden et al., 2013; Speed et al., 2011), and distances between receivers ranged between 0.55 and 4.57 km with mean distance to closest receiver 2.15 km, with minimal overlap between receivers (Appendix 1; Figures S1–S4). We acknowledge that this could lead to periods where sharks remain close to a receiver conducting “restricted” movements without being detected, rather than engaging in wider “out‐of‐range” movements. Unfortunately, due to the logistics of conducting research in the BIOT MPA we were not able to conduct range tests as part of this study to quantify the exact impact this issue may have on our results. We consider the influence of this, however, to be minimal for the following reasons; classification thresholds of movement were very conservative, giving considerable leeway for an animal to move around a reef area, detections to be missed, and still the movement be classed as “restricted”; furthermore, neither of these species are able to rest motionless on the bottom, as they are required to ram ventilate (Skomal et al., 2007). This means these species are less likely to remain in a blind spot for long time periods and less likely for the tag signal to be blocked in the long term by physical objects, therefore increasing the chances of them being detected on the same or additional receivers even when frequenting gaps between or within arrays.

In addition, animals using the lagoons of these atolls would also not necessarily be detected on the array, and movements across the lagoons could also be misdesignated. However, lagoon use in these species tends to be minimal (Barnett et al., 2012; Dale et al., 2011; Economakis & Lobel, 1998) and the isotope signatures obtained from these two species indicate they are not using lagoons for foraging (Curnick et al., 2019). As such, we believe these potential issues should not have impacted our results significantly. However, we stress that this is an inference method being used in lieu of more accurate measurements for large numbers of free‐ranging individuals. It may not be suitable for regions where receivers are spaced further apart, particularly when the study species may exhibit bouts of sedentary behavior, as this could lead to increased chance of missed detections. Simulating the impacts of receiver distance, detection probability and movement classification will be an interesting extension to this work in the future.

Although the consistent nature of our results with those obtained from stable isotope work (Curnick et al., 2019) helps to validate our methodological approach, further validation of classifications for other systems could be carried out using more accurate positional data (e.g., GPS fixes or mark–recapture positions). In our study, although there were several silvertip sharks double‐tagged with both acoustic and PSAT tags, unfortunately the positional data from these satellite tags were not of a high enough resolution to fully confirm our claims. At low latitudes, the error associated with light‐level geolocation estimates from PSAT tags can be very high (Ferreira et al., 2018). For example, the geolocation error from a silvertip shark tagged with a PSAT in the BIOT MPA was estimated at 0.25° or 27.83 km at the equator (Carlisle et al., 2019). In addition, geolocation algorithms used to reconstruct positions from PSAT data only produce a single position per day, which limits their ability to investigate diel differences in location. Although not feasible in this study, the accuracy of this technique should be validated in future studies with more accurate positional data, such as those derived from Fastloc GPS tags (e.g., smart positioning or temperature transmitting tags).

Multiple environmental factors, such as wind, biological noise, and current, can also impact the probability of detections (Kessel et al., 2014; Reubens et al., 2019). In addition, these may differ with diel stage, tides, and lunar cycle, which can lead to reduced detections at night (Payne et al., 2010). Control tests, such as using tags placed in fixed locations, can be carried out to investigate how detection range and probability of detections vary with time of day and different environmental conditions (Payne et al., 2010), and ideally, these tests should be carried out whenever a new acoustic array is set up. For logistical reasons, these control tests have not yet been performed for the BIOT MPA, but it may be feasible to perform these tests in the BIOT MPA in the future to understand the impact of this on our results.

In this study, detection gaps and RDET were combined to have a single metric for both recursive and transitional movement types, in order to detect whether an animal was conducting “restricted” on‐reef movements, or wider, off‐reef and “out‐of‐range” movements. However, there may be differences in recursive and transitional movement types that were not investigated here and using modeling techniques on detection differences and RDET individually would enable further investigations into the movement behavior of fish species. For example, are wider, “out‐of‐range” movements more likely to be undertaken with a recursive or transitional movement? In addition, modeling detection gaps and RDET could be used as response variables separately to further investigate what drives the recursive and transitional movements, respectively.

There are no single “silver bullet” techniques for fully investigating the movement ecology of aquatic species at an appropriate and meaningful spatial and temporal scale; each methodology has its limitations. Here, we show that, in the absence of finer‐scale movement data beyond the boundaries of our acoustic detection ranges, this methodology, using gaps in detections, can be used to investigate, support, and extend conclusions about spatiotemporal movement patterns from acoustic telemetry data, such as how variable behavioral strategies can influence interspecific species organization on coral reef systems. However, there are some limitations that may preclude the use of this approach within other locations/arrays. This approach, however, has the potential to be used to inform understanding of the behavioral biology and ecology of aquatic fauna, particularly in conjunction with other methodologies, principally in regions where high spatial resolution data may not be available, which can assist more informed strategies for the conservation and management of the aquatic environment.

## CONFLICT OF INTEREST

None declared.

## AUTHOR CONTRIBUTIONS

**Michael James Williamson:** Conceptualization (equal); Formal analysis (lead); Methodology (equal); Visualization (lead); Writing‐original draft (lead); Writing‐review & editing (lead). **Emma Tebbs:** Conceptualization (supporting); Supervision (equal); Writing‐review & editing (supporting). **Terence P Dawson:** Writing‐review & editing (supporting). **David J Curnick:** Funding acquisition (equal); Investigation (equal); Supervision (supporting); Writing‐review & editing (supporting). **Francesco Ferretti:** Investigation (equal); Writing‐review & editing (supporting). **Aaron Carlisle:** Investigation (equal); Writing‐review & editing (supporting). **Taylor Chapple:** Investigation (equal); Writing‐review & editing (supporting). **Robert J Schallert:** Investigation (equal). **David Tickler:** Investigation (equal). **Xavier Harrison:** Formal analysis (supporting); Visualization (supporting); Writing‐review & editing (supporting). **Barbara Block:** Funding acquisition (equal); Writing‐review & editing (supporting). **David M.P Jacoby:** Conceptualization (equal); Funding acquisition (equal); Investigation (equal); Methodology (equal); Supervision (equal); Writing‐review & editing (supporting).

## Supporting information

Supplementary Material

## Data Availability

The original acoustic telemetry data used for this analysis are available from the DRYAD Digital Repository: https://doi.org/10.5061/dryad.pg4f4qrmb.
