# Supplementary material for: Analysing detection gaps in acoustic telemetry data to infer differential movement patterns in fish
Source: Ecol Evol. 2021 Feb 10;11(6):2717–30. doi: 10.1002/ece3.7226 (PMC7981221; doi:10.1002/ece3.7226)
Supplement: Supplementary file 1 — Supplementary Material [file ECE3-11-2717-s001.docx]

**Article title:** Temporal resource partitioning in reef sharks confirmed through gap analysis of acoustic tracking data

**Journal name**: Ecology and Evolution

**Author names:** Michael J. Williamson, Emma J. Tebbs, Terence P. Dawson, David J. Curnick, Francesco Ferretti, Aaron B. Carlisle, Taylor K. Chapple, Robert J. Schallert, David M. Tickler, Xavier A. Harrison, Barbara A. Block and David M. P. Jacoby

**Corresponding author**: Michael J. Williamson; Department of Geography, King’s College London, London, [michael.williamson@kcl.ac.uk](mailto:michael.williamson@kcl.ac.uk)

**APPENDIX 1:**


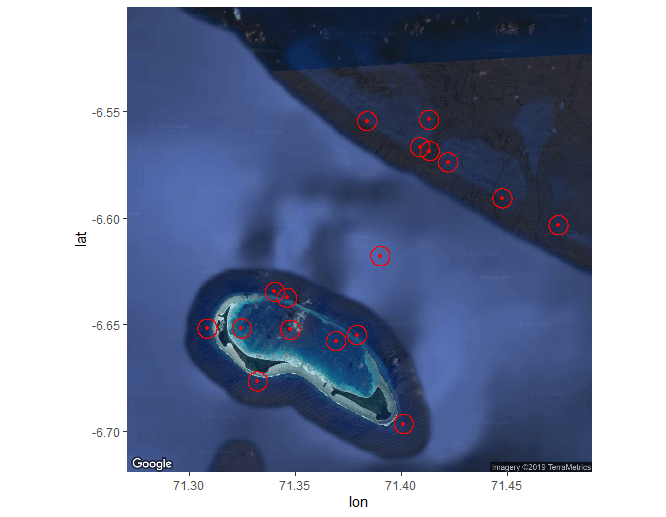
Figure S1 Map of Egmont reef with receiver locations indicated with red dots and a 500m metre buffer of the estimated receiving range around each receiver.


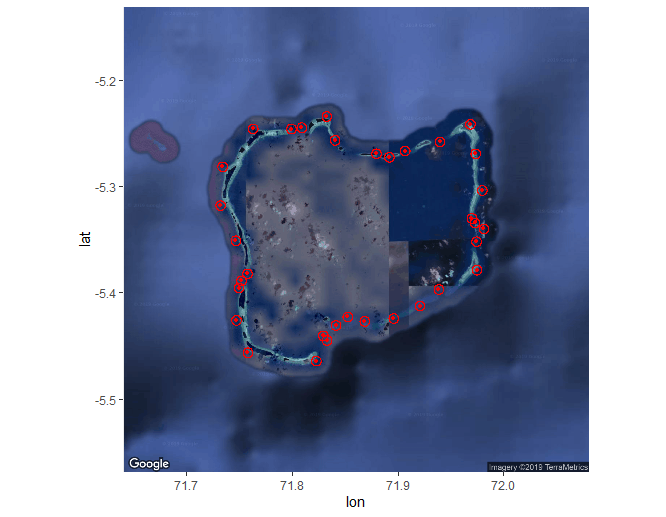


Figure S2 Map of Peros Banos reef with receiver locations indicated with red dots and a 500m metre buffer of the estimated receiving range around each receiver.


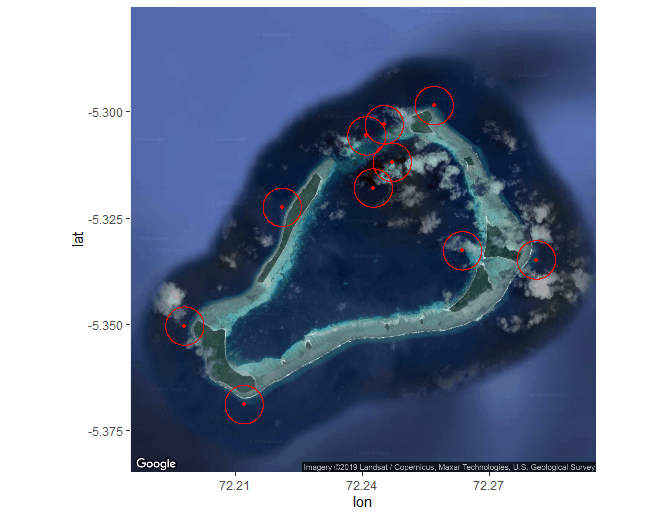


Figure S3 Map of Saloman reef with receiver locations indicated with red dots and a 500m metre buffer of the estimated receiving range around each receiver.


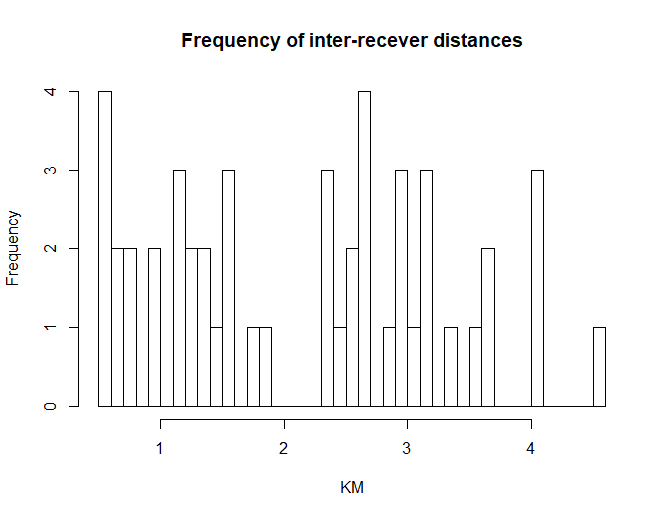


Figure S4 Histogram of inter-receiver distances in the BIOT MP


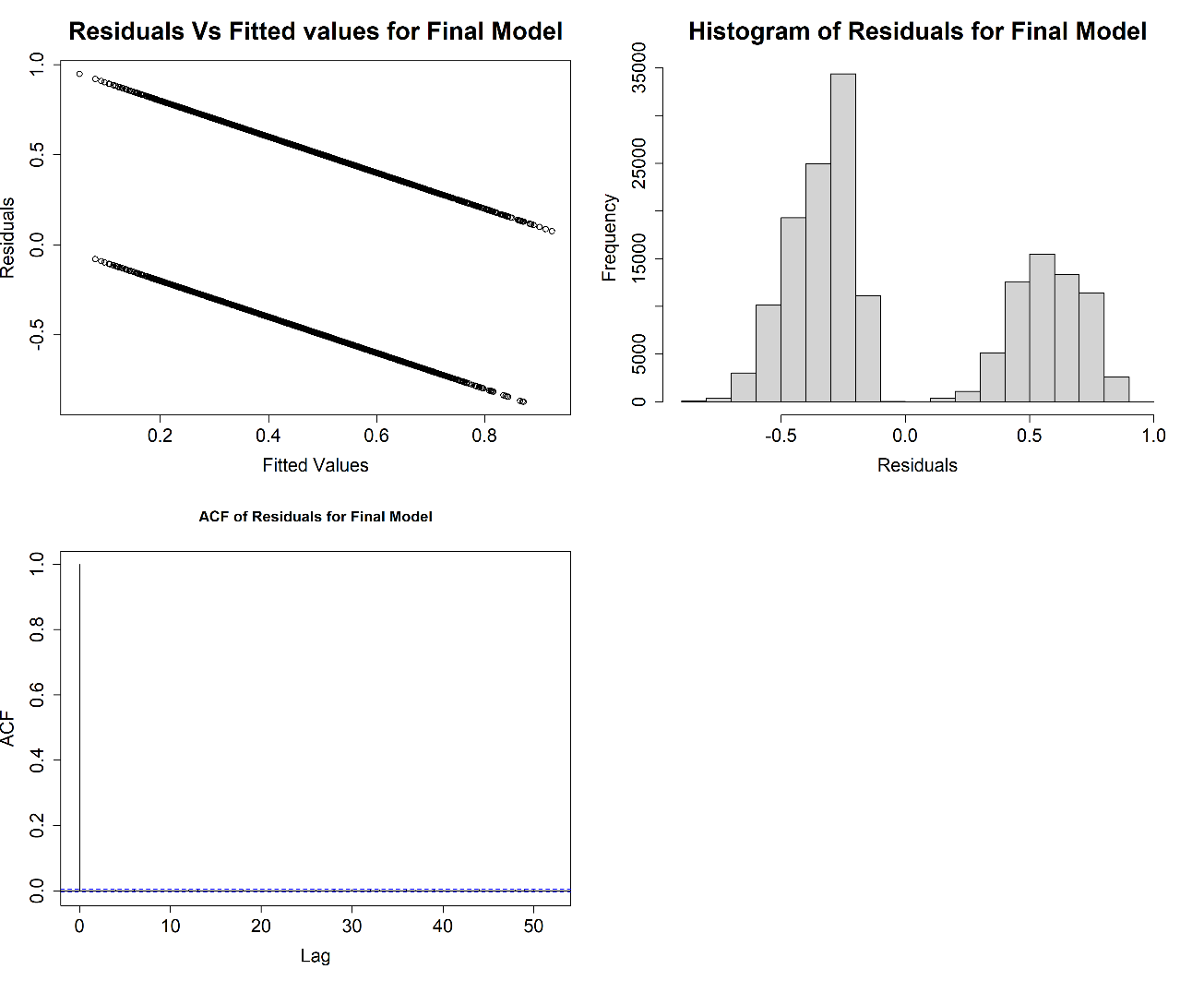


Figure S5 Plots of global model residuals to check for heteroscedasticity, autocorrelation and errors checked for binomial distribution. Binomial distribution of residual vs fitted and histogram of residuals indicate lack of homoscedasticity and a binomial distribution. ACF values indicate no autocorrelation.

Table S1 Metadata for tagged individuals analysed from 2014 to 2018. First detection, last detection, detection duration (in days), sex and size (when tagged), and tagging date are included.

| **ID** | **Species** | **Sex** | **Length** | **Tag date** | **First detection** | **Final detection** | **Detection duration (days)** |
| --- | --- | --- | --- | --- | --- | --- | --- |
| 2388 | Silvertip Shark | F | 112 | 16/04/2016 | 18/06/2016 | 20/09/2017 | 459 |
| 2390 | Grey Reef Shark | M | 118 | 16/04/2016 | 24/04/2016 | 19/09/2016 | 148 |
| 12950 | Silvertip Shark | F | 119 | 25/03/2014 | 01/04/2014 | 03/12/2014 | 246 |
| 12952 | Silvertip Shark | M | 97 | 29/03/2014 | 29/03/2014 | 09/12/2014 | 255 |
| 12956 | Silvertip Shark | F | 150 | 27/03/2014 | 27/04/2014 | 17/06/2014 | 51 |
| 12958 | Silvertip Shark | F | 114 | 27/03/2014 | 03/04/2014 | 03/12/2014 | 244 |
| 12962 | Silvertip Shark | M | 127 | 25/03/2014 | 26/03/2014 | 22/12/2018 | 1732 |
| 12964 | Silvertip Shark | F | 104 | 25/03/2014 | 27/03/2014 | 26/11/2016 | 975 |
| 12966 | Silvertip Shark | M | 117 | 25/03/2014 | 29/03/2014 | 02/11/2018 | 1679 |
| 12968 | Silvertip Shark | M | 115 | 29/03/2014 | 29/03/2014 | 05/12/2014 | 251 |
| 13575 | Grey Reef Shark | F | 94 | 22/03/2013 | 01/01/2014 | 20/03/2018 | 1539 |
| 13576 | Grey Reef Shark | F | 100 | 22/03/2013 | 01/01/2014 | 10/12/2014 | 343 |
| 13577 | Silvertip Shark | M | 119 | 17/03/2013 | 01/01/2014 | 22/01/2017 | 1117 |
| 19318 | Grey Reef Shark | F | 110 | 22/03/2013 | 09/07/2014 | 13/07/2014 | 4 |
| 19506 | Grey Reef Shark | M | 122 | 18/03/2018 | 19/04/2018 | 14/11/2018 | 209 |
| 19514 | Grey Reef Shark | M | 70 | 14/03/2018 | 19/03/2018 | 31/12/2018 | 287 |
| 19516 | Grey Reef Shark | M | 119 | 16/03/2018 | 21/03/2018 | 31/12/2018 | 285 |
| 19517 | Silvertip Shark | F | 129 | 12/04/2016 | 21/12/2016 | 21/12/2016 | 0 |
| 19518 | Grey Reef Shark | F | 125 | 16/03/2018 | 16/03/2018 | 31/12/2018 | 290 |
| 19520 | Grey Reef Shark | F | 121 | 19/03/2018 | 04/07/2018 | 24/09/2018 | 82 |
| 19523 | Silvertip Shark | M | 137 | 16/04/2016 | 26/04/2016 | 18/12/2018 | 966 |
| 19524 | Grey Reef Shark | F | 143 | 16/04/2016 | 17/04/2016 | 11/12/2017 | 603 |
| 25534 | Silvertip Shark | F | 113 | 20/03/2014 | 28/03/2014 | 06/04/2016 | 740 |
| 25535 | Silvertip Shark | M | 108 | 20/03/2014 | 30/03/2014 | 27/04/2014 | 28 |
| 25536 | Silvertip Shark | M | 128 | 22/03/2014 | 02/04/2014 | 05/12/2014 | 247 |
| 25537 | Grey Reef Shark | F | 88 | 20/03/2014 | 21/01/2015 | 31/07/2016 | 557 |
| 25539 | Silvertip Shark | M | 104 | 22/03/2014 | 29/03/2014 | 14/08/2018 | 1599 |
| 25540 | Grey Reef Shark | F | 137 | 22/03/2014 | 01/04/2014 | 11/04/2014 | 10 |
| 25541 | Grey Reef Shark | F | 106 | 22/03/2014 | 30/03/2014 | 13/09/2016 | 898 |
| 25542 | Silvertip Shark | F | 115 | 25/03/2014 | 02/04/2014 | 06/12/2014 | 248 |
| 25543 | Grey Reef Shark | F | 140 | 22/03/2014 | 29/03/2014 | 28/03/2017 | 1095 |
| 25544 | Silvertip Shark | M | 134 | 24/03/2014 | 27/03/2014 | 01/12/2014 | 249 |
| 25545 | Silvertip Shark | F | 108 | 24/03/2014 | 29/03/2014 | 30/09/2017 | 1281 |
| 25546 | Grey Reef Shark | M | 125 | 23/03/2014 | 15/04/2014 | 03/08/2016 | 841 |
| 25547 | Silvertip Shark | F | 118 | 24/03/2014 | 30/03/2014 | 15/07/2014 | 107 |
| 25548 | Silvertip Shark | F | 117 | 25/03/2014 | 27/03/2014 | 10/04/2016 | 745 |
| 25549 | Grey Reef Shark | M | 116 | 23/03/2014 | 30/04/2014 | 25/06/2016 | 787 |
| 25550 | Silvertip Shark | F | 122 | 24/03/2014 | 30/03/2014 | 18/05/2014 | 49 |
| 25552 | Grey Reef Shark | F | 140 | 25/03/2014 | 27/03/2014 | 02/12/2014 | 250 |
| 25553 | Grey Reef Shark | M | 131 | 24/03/2014 | 26/03/2014 | 08/07/2016 | 835 |
| 27584 | Silvertip Shark | F | 108 | 17/03/2013 | 11/01/2014 | 03/12/2014 | 326 |
| 27595 | Grey Reef Shark | F | 97 | 09/02/2013 | 21/05/2016 | 21/05/2016 | 0 |
| 27596 | Grey Reef Shark | F | 114 | 09/02/2013 | 24/04/2014 | 01/06/2014 | 38 |
| 27598 | Silvertip Shark | M | 115 | 13/03/2013 | 05/01/2014 | 10/02/2014 | 36 |
| 27603 | Silvertip Shark | M | 119 | 12/03/2013 | 06/01/2014 | 07/12/2014 | 335 |
| 27605 | Grey Reef Shark | F | 100 | 16/03/2013 | 20/01/2014 | 12/02/2014 | 23 |
| 27606 | Silvertip Shark | F | 107 | 13/03/2013 | 22/01/2014 | 20/02/2014 | 29 |
| 27609 | Silvertip Shark | F | 116 | 12/03/2013 | 01/01/2014 | 27/01/2014 | 26 |
| 27611 | Silvertip Shark | F | 115 | 09/02/2013 | 01/01/2014 | 10/12/2014 | 343 |
| 28608 | Grey Reef Shark | F | 140 | 18/03/2018 | 15/06/2018 | 12/12/2018 | 180 |
| 28611 | Grey Reef Shark | F | 155 | 18/03/2018 | 20/03/2018 | 31/12/2018 | 286 |
| 28612 | Grey Reef Shark | F | 115 | 13/03/2018 | 13/03/2018 | 16/10/2018 | 217 |
| 28618 | Grey Reef Shark | M | 74 | 13/03/2018 | 15/03/2018 | 30/11/2018 | 260 |
| 28624 | Silvertip Shark | F | 110 | 12/03/2018 | 27/11/2018 | 27/11/2018 | 0 |
| 28625 | Grey Reef Shark | F | 107 | 11/03/2018 | 12/03/2018 | 29/10/2018 | 231 |
| 28627 | Silvertip Shark | F | 117 | 12/03/2018 | 07/05/2018 | 07/08/2018 | 92 |
| 28628 | Grey Reef Shark | F | 115 | 11/03/2018 | 21/03/2018 | 23/09/2018 | 186 |
| 28629 | Grey Reef Shark | M | 113 | 11/03/2018 | 13/03/2018 | 29/12/2018 | 291 |
| 28631 | Grey Reef Shark | M | 121 | 11/03/2018 | 12/03/2018 | 09/12/2018 | 272 |
| 28632 | Grey Reef Shark | F | 116 | 11/03/2018 | 12/05/2018 | 29/12/2018 | 231 |
| 28633 | Grey Reef Shark | F | 82 | 13/03/2018 | 17/03/2018 | 28/10/2018 | 225 |
| 28637 | Silvertip Shark | M | 124 | 12/03/2018 | 29/04/2018 | 04/08/2018 | 97 |
| 28638 | Grey Reef Shark | F | 86 | 11/03/2018 | 04/11/2018 | 31/12/2018 | 57 |
| 28639 | Silvertip Shark | F | 94 | 12/03/2018 | 18/04/2018 | 06/06/2018 | 49 |
| 28641 | Grey Reef Shark | M | 95 | 11/03/2018 | 12/03/2018 | 21/12/2018 | 284 |
| 28642 | Silvertip Shark | M | 103 | 13/03/2018 | 13/03/2018 | 10/10/2018 | 211 |
| 28644 | Grey Reef Shark | F | 132 | 18/03/2018 | 01/04/2018 | 20/04/2018 | 19 |
| 28660 | Silvertip Shark | F | 125 | 19/03/2018 | 21/03/2018 | 10/11/2018 | 234 |
| 28662 | Grey Reef Shark | F | 133 | 19/03/2018 | 20/03/2018 | 29/12/2018 | 284 |
| 28670 | Silvertip Shark | M | 125 | 19/03/2018 | 23/03/2018 | 29/12/2018 | 281 |
| 28672 | Grey Reef Shark | M | 112 | 19/03/2018 | 20/03/2018 | 31/12/2018 | 286 |
| 28674 | Grey Reef Shark | F | 123 | 19/03/2018 | 20/03/2018 | 31/12/2018 | 286 |
| 34176 | Silvertip Shark | F | 140 | 22/03/2013 | 07/01/2014 | 21/11/2014 | 318 |
| 34177 | Silvertip Shark | M | 115 | 22/03/2013 | 01/01/2014 | 08/12/2014 | 341 |
| 34178 | Silvertip Shark | F | 102 | 22/03/2013 | 27/07/2014 | 27/07/2014 | 0 |
| 34179 | Silvertip Shark | F | 99.5 | 17/03/2013 | 04/01/2014 | 03/03/2014 | 58 |
| 52975 | Silvertip Shark | M | 123 | 22/03/2013 | 02/01/2014 | 02/12/2014 | 334 |
| 54815 | Grey Reef Shark | F | 118 | 01/04/2016 | 01/04/2016 | 08/04/2018 | 737 |
| 54817 | Silvertip Shark | F | 134 | 01/04/2016 | 04/04/2016 | 24/04/2016 | 20 |
| 54821 | Silvertip Shark | F | 146 | 01/04/2016 | 04/04/2016 | 05/04/2016 | 1 |
| 54822 | Silvertip Shark | F | 135 | 01/04/2016 | 28/04/2016 | 14/10/2016 | 169 |
| 54825 | Silvertip Shark | F | 138.5 | 01/04/2016 | 06/04/2016 | 06/04/2016 | 0 |
| 54845 | Silvertip Shark | F | 151 | 12/04/2016 | 26/09/2016 | 01/10/2016 | 5 |
| 54846 | Grey Reef Shark | F | 135 | 08/04/2016 | 09/04/2016 | 31/12/2018 | 996 |
| 54849 | Silvertip Shark | M | 137 | 08/04/2016 | 10/04/2016 | 22/12/2018 | 986 |
| 54851 | Grey Reef Shark | F | 171 | 10/04/2016 | 07/06/2016 | 09/06/2018 | 732 |
| 54852 | Grey Reef Shark | M | 114 | 08/04/2016 | 09/04/2016 | 31/12/2018 | 996 |
| 54858 | Silvertip Shark | M | 112 | 10/04/2016 | 11/05/2016 | 14/06/2016 | 34 |
| 54860 | Grey Reef Shark | F | 136 | 07/04/2016 | 07/04/2016 | 27/04/2016 | 20 |
| 54863 | Grey Reef Shark | F | 156 | 08/04/2016 | 09/04/2016 | 09/04/2016 | 0 |
| 54864 | Grey Reef Shark | F | 120 | 10/04/2016 | 10/04/2016 | 28/11/2018 | 962 |
| 54866 | Grey Reef Shark | F | 141 | 08/04/2016 | 28/04/2016 | 31/12/2018 | 977 |
| 54868 | Grey Reef Shark | M | 106 | 08/04/2016 | 22/03/2018 | 19/04/2018 | 28 |
| 54869 | Grey Reef Shark | F | 127 | 10/04/2016 | 29/04/2016 | 13/04/2017 | 349 |
| 54872 | Grey Reef Shark | F | 118 | 10/04/2016 | 11/04/2016 | 13/07/2016 | 93 |
| 54874 | Grey Reef Shark | F | 97 | 10/04/2016 | 25/04/2016 | 14/06/2016 | 50 |
| 54877 | Grey Reef Shark | F | 143 | 08/04/2016 | 18/05/2016 | 02/11/2016 | 168 |
| 54880 | Silvertip Shark | F | 87 | 15/04/2016 | 26/04/2016 | 30/12/2018 | 978 |
| 54882 | Grey Reef Shark | M | 134 | 15/04/2016 | 20/04/2016 | 30/08/2016 | 132 |
| 54883 | Grey Reef Shark | F | 100 | 15/04/2016 | 12/06/2016 | 30/08/2018 | 809 |
| 54884 | Grey Reef Shark | F | 155 | 16/04/2016 | 20/04/2016 | 01/09/2016 | 134 |
| 54886 | Grey Reef Shark | F | 137 | 15/04/2016 | 19/04/2016 | 11/01/2018 | 632 |
| 54887 | Silvertip Shark | F | 161 | 16/04/2016 | 17/04/2016 | 28/11/2017 | 590 |
| 54889 | Silvertip Shark | F | 88 | 15/04/2016 | 21/04/2016 | 31/12/2018 | 984 |
| 54890 | Grey Reef Shark | M | 136 | 16/04/2016 | 16/04/2016 | 23/04/2018 | 737 |
| 54892 | Grey Reef Shark | F | 132 | 15/04/2016 | 17/06/2016 | 01/11/2017 | 502 |
| 54893 | Silvertip Shark | F | 162 | 15/04/2016 | 18/04/2016 | 29/10/2018 | 924 |
| 54895 | Grey Reef Shark | M | 106 | 15/04/2016 | 15/04/2016 | 14/12/2018 | 973 |
| 54896 | Grey Reef Shark | F | 157 | 15/04/2016 | 21/08/2016 | 09/12/2017 | 475 |
| 54898 | Silvertip Shark | M | 151 | 12/04/2016 | 10/10/2016 | 29/12/2018 | 810 |
| 54899 | Silvertip Shark | M | 203 | 14/04/2016 | 16/04/2016 | 27/10/2017 | 559 |
| 54901 | Grey Reef Shark | F | 152 | 14/04/2016 | 17/05/2016 | 09/10/2018 | 875 |
| 54904 | Grey Reef Shark | F | 122 | 14/04/2016 | 18/04/2016 | 16/09/2018 | 881 |
| 54905 | Silvertip Shark | F | 120 | 14/04/2016 | 22/04/2016 | 22/06/2016 | 61 |
| 54911 | Silvertip Shark | F | 104 | 14/04/2016 | 19/07/2016 | 11/12/2018 | 875 |
| 54912 | Silvertip Shark | M | 196 | 15/04/2016 | 29/05/2016 | 04/06/2018 | 736 |
| 54914 | Grey Reef Shark | F | 117 | 16/04/2016 | 25/04/2016 | 31/12/2018 | 980 |
| 54915 | Silvertip Shark | F | 139 | 16/04/2016 | 18/04/2016 | 18/12/2018 | 974 |
| 54919 | Grey Reef Shark | F | 118 | 14/03/2018 | 20/03/2018 | 31/12/2018 | 286 |
| 54920 | Grey Reef Shark | M | 138 | 18/03/2018 | 05/04/2018 | 24/12/2018 | 263 |
| 54922 | Grey Reef Shark | F | 132 | 14/03/2018 | 24/03/2018 | 31/12/2018 | 282 |
| 54925 | Grey Reef Shark | F | 135 | 14/03/2018 | 19/03/2018 | 31/12/2018 | 287 |
| 54927 | Grey Reef Shark | F | 86 | 16/03/2018 | 17/03/2018 | 31/12/2018 | 289 |
| 54929 | Grey Reef Shark | F | 108 | 16/03/2018 | 05/10/2018 | 05/10/2018 | 0 |
| 54931 | Grey Reef Shark | M | 121 | 15/04/2016 | 18/04/2016 | 31/12/2018 | 987 |
| 54932 | Grey Reef Shark | M | 135 | 14/03/2018 | 19/03/2018 | 31/12/2018 | 287 |
| 54933 | Silvertip Shark | F | 148 | 14/04/2016 | 15/04/2016 | 26/12/2018 | 985 |
| 54938 | Grey Reef Shark | F | 148 | 14/03/2018 | 26/03/2018 | 31/12/2018 | 280 |
| 54947 | Silvertip Shark | F | 144 | 14/04/2016 | 27/04/2016 | 31/12/2018 | 978 |
| 54949 | Grey Reef Shark | M | 128 | 15/03/2018 | 29/03/2018 | 03/11/2018 | 219 |
| 54952 | Grey Reef Shark | F | 146 | 23/03/2016 | 23/03/2016 | 21/09/2018 | 912 |
| 54954 | Grey Reef Shark | F | 145 | 24/03/2016 | 06/04/2016 | 30/09/2018 | 907 |
| 54955 | Grey Reef Shark | F | 142 | 23/03/2016 | 28/03/2016 | 30/09/2018 | 916 |
| 54957 | Silvertip Shark | F | 93 | 24/03/2016 | 25/03/2016 | 26/09/2018 | 915 |
| 54958 | Grey Reef Shark | F | 138 | 23/03/2016 | 07/04/2016 | 25/12/2018 | 992 |
| 54959 | Grey Reef Shark | F | 104 | 23/03/2016 | 23/03/2016 | 29/09/2016 | 190 |
| 54961 | Silvertip Shark | M | 86 | 23/03/2016 | 25/03/2016 | 29/12/2018 | 1009 |
| 54962 | Grey Reef Shark | F | 117 | 24/03/2016 | 24/03/2016 | 22/10/2018 | 942 |
| 59950 | Grey Reef Shark | F | 110 | 22/03/2015 | 26/03/2015 | 20/09/2017 | 909 |
| 59951 | Silvertip Shark | M | 183 | 22/03/2015 | 17/04/2015 | 25/06/2016 | 435 |
| 59953 | Grey Reef Shark | M | 110 | 22/03/2015 | 12/03/2016 | 12/05/2017 | 426 |
| 59955 | Grey Reef Shark | M | 131 | 22/03/2015 | 03/05/2015 | 19/05/2015 | 16 |
| 59957 | Grey Reef Shark | F | 133 | 21/03/2015 | 06/04/2015 | 09/10/2018 | 1282 |
| 59958 | Grey Reef Shark | F | 141 | 21/03/2015 | 24/03/2015 | 11/12/2018 | 1358 |
| 59959 | Grey Reef Shark | F | 133 | 21/03/2015 | 01/05/2015 | 18/11/2017 | 932 |
| 59960 | Grey Reef Shark | F | 150 | 21/03/2015 | 17/06/2015 | 03/10/2015 | 108 |
| 59961 | Grey Reef Shark | F | 145 | 21/03/2015 | 08/04/2015 | 19/09/2018 | 1260 |
| 59962 | Grey Reef Shark | F | 136 | 21/03/2015 | 19/03/2015 | 13/09/2018 | 1274 |
| 59963 | Silvertip Shark | F | 120 | 19/03/2015 | 19/03/2015 | 28/12/2018 | 1380 |
| 59964 | Grey Reef Shark | F | 112 | 19/03/2015 | 19/03/2015 | 01/11/2017 | 958 |
| 59966 | Grey Reef Shark | F | 139 | 0019-03-15 | 27/03/2015 | 27/03/2015 | 0 |
| 59968 | Silvertip Shark | M | 178 | 22/03/2015 | 04/04/2015 | 03/02/2016 | 305 |
| 59969 | Grey Reef Shark | F | 105 | 23/03/2015 | 01/04/2015 | 14/01/2017 | 654 |
| 59970 | Grey Reef Shark | M | 125 | 23/03/2015 | 07/04/2015 | 14/10/2018 | 1286 |
| 59971 | Grey Reef Shark | F | 97 | 23/03/2015 | 23/03/2015 | 28/08/2017 | 889 |
| 59972 | Grey Reef Shark | F | 101 | 23/03/2015 | 23/03/2015 | 12/04/2016 | 386 |
| 59973 | Grey Reef Shark | F | 70 | 23/03/2015 | 10/04/2015 | 05/11/2017 | 940 |
| 59975 | Grey Reef Shark | F | 114 | 23/03/2015 | 25/03/2015 | 12/04/2016 | 384 |
| 59976 | Grey Reef Shark | M | 106 | 23/03/2015 | 27/03/2015 | 31/12/2018 | 1375 |
| 59978 | Silvertip Shark | F | 166 | 23/03/2015 | 23/04/2015 | 13/06/2016 | 417 |
| 59979 | Grey Reef Shark | F | 96 | 23/03/2015 | 14/04/2015 | 15/12/2018 | 1341 |
| 59980 | Grey Reef Shark | F | 73 | 24/03/2015 | 16/04/2015 | 18/07/2018 | 1189 |
| 59981 | Grey Reef Shark | M | 131 | 23/03/2015 | 25/03/2015 | 21/09/2016 | 546 |
| 59982 | Silvertip Shark | M | 109 | 25/03/2015 | 30/03/2015 | 03/12/2016 | 614 |
| 59984 | Silvertip Shark | M | 134 | 25/03/2015 | 10/04/2015 | 28/04/2017 | 749 |
| 59985 | Silvertip Shark | M | 123 | 25/03/2015 | 26/04/2015 | 26/04/2017 | 731 |
| 59986 | Silvertip Shark | F | 127 | 25/03/2015 | 26/03/2015 | 27/03/2017 | 732 |
| 59987 | Silvertip Shark | M | 181 | 25/03/2015 | 03/06/2015 | 14/04/2017 | 681 |
| 59988 | Grey Reef Shark | F | 112 | 27/03/2015 | 27/03/2015 | 05/12/2018 | 1349 |
| 59991 | Silvertip Shark | F | 132 | 28/03/2015 | 14/04/2015 | 30/12/2018 | 1356 |
| 59992 | Silvertip Shark | M | 131 | 27/03/2015 | 01/04/2015 | 14/04/2018 | 1109 |
| 59993 | Silvertip Shark | M | 157 | 27/03/2015 | 03/04/2015 | 31/12/2018 | 1368 |
| 59994 | Silvertip Shark | F | 153 | 27/03/2015 | 05/04/2015 | 03/04/2016 | 364 |
| 59995 | Silvertip Shark | F | 143 | 27/03/2015 | 03/04/2015 | 05/03/2016 | 337 |
| 59996 | Grey Reef Shark | F | 124 | 25/03/2015 | 29/03/2015 | 28/04/2017 | 761 |
| 59997 | Grey Reef Shark | F | 108 | 25/03/2015 | 21/11/2015 | 09/04/2017 | 505 |
| 59999 | Grey Reef Shark | F | 122 | 25/03/2015 | 26/03/2015 | 04/10/2018 | 1288 |

Table S2 Outputs from collinearity tests using ‘check_collinearity’ function in the *performance* package.

| **Predictor** | **VIF** | **SE_factor** |
| --- | --- | --- |
| species | 1.046700 | 1.023083 |
| sex | 1.038251 | 1.018946 |
| log (size) | 1.008352 | 1.000041 |
| diel stage | 1.000041 | 1.000020 |
| season | 1.000055 | 1.000027 |
